# “Whom Should I Pass To?” The More Options the More Attentional Guidance from Working Memory

**DOI:** 10.1371/journal.pone.0062278

**Published:** 2013-05-02

**Authors:** Philip Furley, Daniel Memmert

**Affiliations:** German Sport University, Cologne, Germany; Universidad de Granada, Spain

## Abstract

Three experiments investigated the predictions of the biased competition theory of selective attention in a computer based sport task. According to this theory objects held in the circuitry of working memory (WM) automatically bias attention to objects in a visual scene that match or are related to the WM representation. Specifically, we investigated whether certain players that are activated in the circuitry of WM automatically draw attention and receive a competitive advantage in a computer based sport task. In all three experiments participants had to hold an image of a certain player in WM while engaged in a speeded sport task. In Experiment 1 participants had to identify as quickly as possible which player was in possession of the ball. In Experiment 2 and 3 participants had to decide to which player they would pass to in a cartoon team handball situation and a photo picture basketball situation. The results support the biased competition theory of selective attention and suggest that certain decision options receive a competitive advantage if they are associated with the activated contents in the circuitry of WM and that this effect is more pronounced when more decision options compete for attention. A further extension compared to previous research was that the contents of working memory not only biased attention but also actual decisions that can lead to passing errors in sport. We critically discuss the applied implications of the findings.

## Introduction

Team sport athletes need to be able to quickly and efficiently select situation appropriate actions under extreme time pressure in high interference situations: for example when a quarterback in American football tries to find an open receiver in the final offensive play or a point guard in basketball has to make a split second decision of whom to pass the ball to. Given the endless amount of information that bombards players of team ball games it is often not possible for a player to consider all the tactical decision-making possibilities in highly time constrained situations in which numerous team-members and opponents compete for limited attentional resources. Thus, players often form prior intentions of whom to pass to or the coach gives tactical instructions and practices offensive plays in the hope of assisting the tactical decision making process. Recent research [1], [2], [3] suggests that attention and working memory (WM) [4], [5] are key cognitive factors for understanding performance in these situations. A cognitive theory emphasizing a close interaction between attention and WM that might account for the mental processes in the aforementioned sport situations is the biased competition theory of selective attention [6]. In the present research we test the predictions derived from the biased competition theory of selective attention in a computer simulated sports task as sports offers an suitable context due to its situational constraints–for example time pressure; multiple team-members and opponents–imposed on the athletes.

### The Biased Competition Theory of Selective Attention (BCT)

According to information processing accounts of human behavior, visual objects in the world compete for cognitive representation, analysis, and control sometime between stimulus input and response as our sensory system cannot process all the available information [7], [8], [9]. In a nutshell, the theory [6] proposes that attention serves to enhance the response of behaviorally relevant neurons and that the effect of attention on neuronal responses is best understood as competition between competing stimuli and representations [10]. Specifically, BCT suggests that top-down control is influenced by an internal template activated in the circuitry of WM, priming an object in the visual scene at the disadvantage of competing objects in the same visual scene [11]. For example, stronger sensory inputs usually have an advantage over weaker sensory stimuli, but the content of WM can bias the competition, tipping the balance towards the weaker stimuli. Hence, if a visual object is preactivated in WM and later appears in the visual display, this object will have an advantage in the competition for selective attention. The winner of this competition then becomes the focus of attention.

Evidence for BCT shows that the contents of WM influence the guidance of selective attention [12]–[18] by modulating the sensitivity of neural circuits that represent the information which improves the signal-to-noise ratio in favour of the information currently being processed in WM [10], [19]. In a typical paradigm [15], [17], [18] that has been utilized to provide evidence for BCT participants have to memorize a certain shape and subsequently search for a tilted line in a search display comprised of various shapes. When the tilted line falls within the memorized shape (valid) search is usually fastest compared to when the shape is not present in the search display (neutral), and slowest if the shape is present but does not contain the tilted target (invalid) (see [20] for a similar paradigm). A further study [13] comprised a speeded two-alternative decision task after holding an item in WM instead of a visual search task. Results provided evidence for attentional guidance from WM when there was neither an explicit search goal nor was the memory task obviously related with the speeded decision task (see also [21] for a similar paradigm).

### Attentional Research Paradigms in Explaining Everyday Behavior

Recently, attentional research paradigms have been criticized for having lost sight of real world behavior and that some of the most prominent research paradigms in the study of attention “run the serious risk of excluding the exploration of questions that are crucial to a fuller understanding of human attention and behavior” [22] (p. 179). In this respect, Kingstone and colleagues [22] demonstrated that a small alteration to a seminal spatial cuing paradigm [23] that replaced predictive arrows (pointing either to the left or right) of the original paradigm by predictive gaze behavior (schematic faces looking either to the left or right) dramatically altered the pattern of findings. This challenged the apparently sound conclusion based on numerous studies that had exclusively used the original paradigm–that a central directional stimulus must be spatially predictive to result in a spatial shift of attention. Of particular relevance to the present research, Kingstone and colleagues [22] state that this observation does not only account for the Posner [24] cuing paradigm but that “the same conclusion holds for many other laboratory paradigms used to study attention, such as the visual search paradigm” (p. 179).

Thus, it is currently not clear how far reaching BCT theory is and whether the findings might only be due to highly specific laboratory paradigms and stimuli. A recent review of studies on BCT [16] specifies this argumentation for attentional guidance effects from WM: “Future studies also need to examine the ecological constraints of the influence of WM on selection, for example, assessing effects in more real life environments” (p. 346). Therefore, the predictive and explanatory range of a theory has to be extended to different paradigms and stimuli in order to provide converging evidence for the underlying concepts in question and demonstrate a theory’s universality [25]. One suggested remedy in this endeavor is creating tasks and stimuli that are grounded in the real world [22] and systematically compare advocated cognitive mechanisms and behavior at different levels of abstraction [26]. In a recent review advocating this approach Risko and colleagues [26] state that “the purpose of the present review is not to espouse a particular direction (i.e., from artificial to naturalistic versus naturalistic to artificial) but rather to champion the act of moving along that continuum in either direction” (p. 8). Hence, we attempt to transfer BCT to a computer based sports task that is grounded in the real life sport situation of e.g. a basketball point guard making a passing decision after having formed the intention to pass to a certain player–which we argue involves holding a representation of that player in WM.

### The Present Research

In the present research we followed the call of Soto and colleagues [16] of examining attentional guidance effects of WM in more real life environments whilst remaining a high level of experimental control. In this endeavor we addressed two specific questions raised by Soto et al. [16]: (a)”Do effects of WM guidance emerge even as the complexity of the environment increases?; (b) “Can the automatic capture of attention by WM lead to some of the action errors that can occur in everyday situations?” (p. 346).

In terms of question (a) sport affords researchers with an suitable context to test BCT in a experimental context grounded in the real world [22] because sports typically involve time pressured dynamic conditions in which athletes have to employ attention efficiently in order to select one out of several decision options, for example which team-member to pass to. In addition almost all of the visual objects, such as team-members and opponents are behaviorally relevant and compete for limited attentional resources. The following example illustrates a common observation in basketball which might be accounted for by the cognitive mechanisms proclaimed by BCT: a basketball point guard might not pass to a team-member under the “hoop” who is waving (stronger stimulus) but instead passes to the shooting guard at the three point line because of the intended offensive play announced by the coach during the last timeout, in which he was told that the team needs open 3-point shots in order to win the game. In this scenario the point guard is probably holding a representation of the player he is attempting to pass the ball to in his or her WM. According to BCT this representation is likely to bias attention towards that player and in turn increase the chances of passing the ball to this player. If this player is unmarked, then the activated template of that player will facilitate the decision to pass to him. On the other hand, if the player is guarded by an opposing player, attention will still be allocated towards that player and will have to be reallocated towards an open team-member, which will consume valuable milliseconds of the limited time available in fast moving sports. By grounding our experimental paradigm in this real world sport example we initiate to extend BCT along the continuum from “artificial to naturalistic” contexts [26].

This brings us directly to question (b), whether the automatic capture of attention by WM might account for some of the frequently observed incidences when for example a basketball player does not pass to obviously unmarked team-members and instead chooses a different passing option as the contents of WM might not only bias the allocation of attention but also the actual behavioral response of passing to a certain player. Hence, we examined whether the contents of WM have the potential of affecting passing decisions which might result in passing to a guarded player if he or she matched a representation that was currently being held in WM. Currently, little is known about attentional guidance effects if the visual objects competing for attention require different actions as would be the case in selecting a passing option in sport. Previous studies usually required a discrimination task at different spatial locations using identical key presses regardless of object location [16], [27] and it remains unclear how WM and attention interact if one has to decide between several objects competing for attention and action as is the case in various everyday situations.

Of further theoretical relevance to question (a) ambivalent predictions and equivocal findings exist concerning the attentional guidance effect of WM as a function of search set size [15], [27] with search set size being operationalized as the number of potential search targets and distractors. It has been suggested [28], [29] that attention reduces its spatial window in tasks with a large set size due to a more effortful search mode. Hence, Olivers [27] theorized that increasing the number of objects present in the stimuli display would result in a narrower attentional window as participants adopt a more effortful, serial search mode. This narrow attentional window in turn should result in ignoring many visual objects including the potential memory matching object and therefore result in a decrease or even total absence of an attentional guidance effect from WM. The results [27] did not support this theory as similar attentional guidance effects from WM were evident in both the small set size and large set size condition which led him to conclude that task set size does little to change the working memory-attention interaction. In contrast Soto et al. [15] found precisely the opposite as predicted by Olivers [27]: a greater attentional guidance effect from WM as a function of more objects in the visual display. An important study [30] that experimentally induced either a narrow or a broad attentional window reported greater attentional guidance effects from WM when participants adopted a broad attentional window. The authors concluded that having a broad attentional window increases the probability that attention will be captured by an object in the display matching a representation being held in WM. Thus, it seems feasible that increasing the number of objects may not result in a narrow attentional window as hypothesized by Olivers [27]. On the contrary, increasing the set size seemed to have resulted in an increase of the attentional window and in turn to greater attentional guidance effects.

Clearly, more research is warranted to further illuminate the effects of set size in the search task on attentional guidance from WM. Again, team sports offers a suitable domain in this endeavor as previous research has suggested that successful team-sports decision making in a computer based sports task requires a broad attentional focus [31] to incorporate all promising passing options and selecting the best one. Therefore, we expect that increasing the potential targets competing for limited attentional capacities in a computer based team-sport task will also result in a broadening of attention and therefore result in greater attentional guidance effects.

Finally, we explored experience related differences in team-sport decision making on attentional guidance effects from WM, as it seems feasible that experienced athletes might be differently affected by attentional guidance effects as a consequence of their extensive experience, even in a computer based sports task that schematically models specific sport situations. Although the majority of findings on sports expertise would not suggest experienced based differences on such tasks as utilized in the present series of studies [32], it has recently been suggested that experienced athletes might also be experts in the cognitive laboratory [33].

## Experiment 1: “Who’s Got the Ball?”

**Figure 1:**
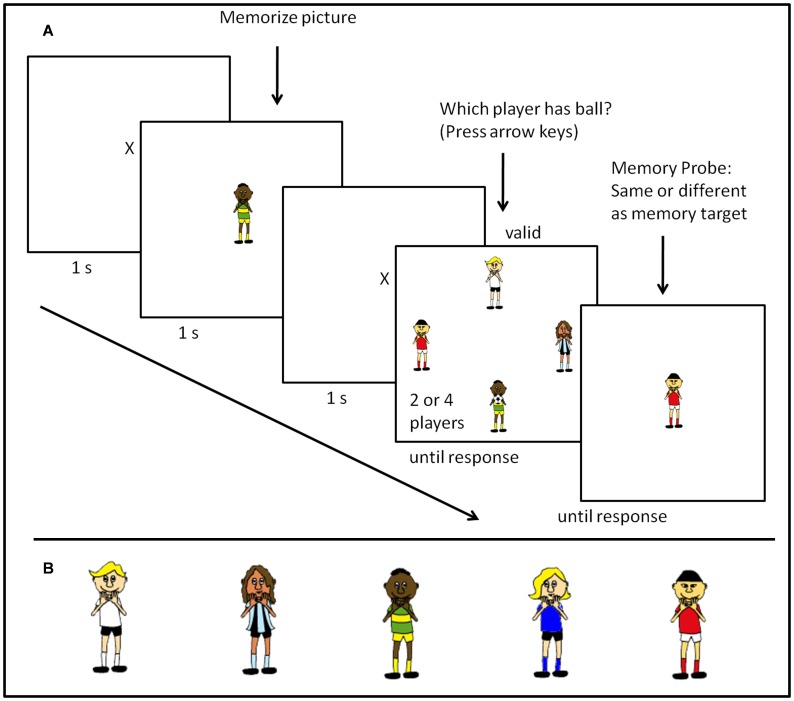
Task and stimuli utilized in Experiment 1. (A) Sequence of a sample trial in Experiment 1. (B) Cartoon characters from the easy Sport-Graphic Software 2.0 Handball used as memory items in Experiment 1.

The aim of Experiment 1 was to replicate previous findings using a different task and novel stimulus material–which is currently a “hot topic in psychological science” [34], [35]. In addition, Experiment 1 was a first step to transfer BCT to the field of sport. In this endeavor we took cartoon figures (Figure 1) from the Easy Sports-Graphics Software and asked participants to identify which player is in possession of the ball after having memorized a certain cartoon figure. The experimental task in Experiment 1 was derived from previous research [13]–[15], [17], [18] and experimentally manipulated the validity of the memory item and the task set size. The theoretical rationale of memory item validity was to examine differences in search response times and accuracy as a function of the activated templates in WM which is an established method in cognitive psychology to test the predictions of BCT. The rationale for the different task set size was derived from the ambiguous findings reviewed above and attempted to clarify the moderating effect of task set size on the top-down guidance of attention from WM by including varying numbers of behaviourally relevant objects competing for attention in the visual search display.

Based on BCT we hypothesized that participants (a) would detect the ball fastest if the player being held in WM was in possession of the ball (valid trials) and slowest if the player being held in WM appeared in the display but was not in possession of the ball (invalid trial) as attention would be automatically drawn towards an object in the visual field that matched the content of WM. Therefore, response times should fall in between valid and invalid trials if the object being held in WM is not present in the visual display (neutral trials) and therefore cannot draw attention. Further, we hypothesized (b) that by increasing the number of potential “ball-holders” in the experimental task participants would be required to increase their attentional window in order to incorporate all potential targets [30] which in turn would increase the probability of attention being captured by an memory-matching object. Thus, attentional guidance effects should interact with number of potential targets as validity effects would be more pronounced the more objects compete for attention. In addition, we explored attentional guidance effects as a function of team-sport experience. We did not expect to find an effect of team-sport experience as the task studied was not representative of the domain specific experience of team sport athletes. However, some research groups have suggested that athletes might perform better in attentional research paradigms [33] compared to a non-athlete control group.

### Method

#### Ethics statement

The study was approved by the Ethics board of the German Sport University Cologne. Informed consent was obtained from every participant before commencing the experiment. The study was carried out in accordance with the Helsinki Declaration of 1975. Before commencing the experiment participants filled out a questionnaire gathering biographic data.

#### Participants

Altogether 24 adults (18 male and 6 female; age: *M* = 25.25) took part in the study. Neither age nor gender moderated the pattern of results in Experiment 1. Half of the participants (*n* = 12) were experienced Team Handball players (*M* = 12.21 years of competitive handball experience), whereas the other half had no competitive team-sports experience. The participants were unaware of the purpose of the study. All of the participants reported to have normal or corrected to normal vision. The participants volunteered to participate in the study and no kind of compensation was given for participation in the study.

#### Apparatus

The experiment was run on an Intel core 2 duo laptop with a screen size of 15.4 inch with the monitor resolution set to 1,024×768 pixels. The task was programmed with E-Prime Professional [36]. The frame rate was fixed at 60 Hz.

#### Task and stimuli

Figure 1 gives a schematic illustration of a sample trial of Experiment 1. Each experimental trial started with a fixation cross being presented for 1500 ms followed by the presentation of the visual memory item (cf. Figure 1B)–a uniquely dressed cartoon figure from the easy Sports-Graphics 2.0 Handball software–for 1000 ms. Participants were instructed to memorize the cartoon figure as they would be asked to identify it in a subsequent memory probe task. Responses in the memory task were made by pressing the “c” key for the same and the “n” key for different. It was the same in 50% of the trials and different in the remaining 50%. Another fixation cross appeared for 1500 ms after the memory item was presented, fixating the gaze of the participants exactly on the centre of the subsequent search display. Depending on the experimental block the search display comprised an array of either two or four cartoon characters from the easy Sports-Graphics Software and was presented until a response was given by the participants. The cartoon characters were positioned around an imaginary clock face of 13.3° radius and occupied the 12, 3, 6, and 9 o’clock position in the 4 player condition, whereas in the two player condition the cartoon figures where arranged either horizontally–3 and 9 o’clock–or vertically–12 and 6 o’clock. The cartoon figures size was 4.8°×1.9° radius in both the memory and decision displays. The participants’ task was to identify as quickly and accurately as possible which of the either two or four players was holding a ball by pressing the corresponding arrow keys.

#### Procedure

In the first phase of the experiment, participants were familiarized with the following procedure with a block of 12 practice trials. The practice block used exactly the same procedure as the following experimental block. Both experimental blocks–set size 2 or set size 4–consisted of 72 trials. The order of blocks was random. Both the memory object and the players in the decision array were selected randomly from the five cartoon figures displayed in Figure 1B. The ratio of valid, neutral, and invalid trials was 33 per cent in both the practice and experimental blocks. On valid trials the memory item was the same as the ball holder in the decision array. On neutral trials the memory item was not present in the decision array. On invalid trials the memory item was present in the decision array, but was not in possession of the ball. Participants were instructed to respond as quickly and as accurately as possible on the decision task, whereas only accuracy was emphasized on the memory probe task.

#### Data analysis

There was neither a main effect nor any interactions (all *p*>.34) for the factor handball experience on both RTs and errors and we therefore did not further differentiate between team handball experience in Experiment 1. We ran two-factor univariate analysis of variance (ANOVA) with repeated measures on both within subject independent variables (validity and set size) on both response times and decision errors. As decision errors were scarce and the distribution was skewed we transformed the error data to the logarithm to the base 10 in order to avoid overestimation of significance values as suggested by an anonymous reviewer. As no errors were made by several participants in experimental categories and it is not possible to log transform 0 we added the percentage corresponding to one error to the error data in every experimental category and log transformed this value. Where, the assumption of sphericity was violated, the p-values were computed using the conservative Greenhouse-Geisser method with corrected degrees of freedom. We followed up significant main effects and interactions with planned contrasts.

### Results

Performance on the memory task was good (*M* = 96.17% correct, *SD* = 2.5) and demonstrated that participants were holding the object in WM which is essential for testing the predictions of BCT.

#### Response times (RT)

**Figure 2:**
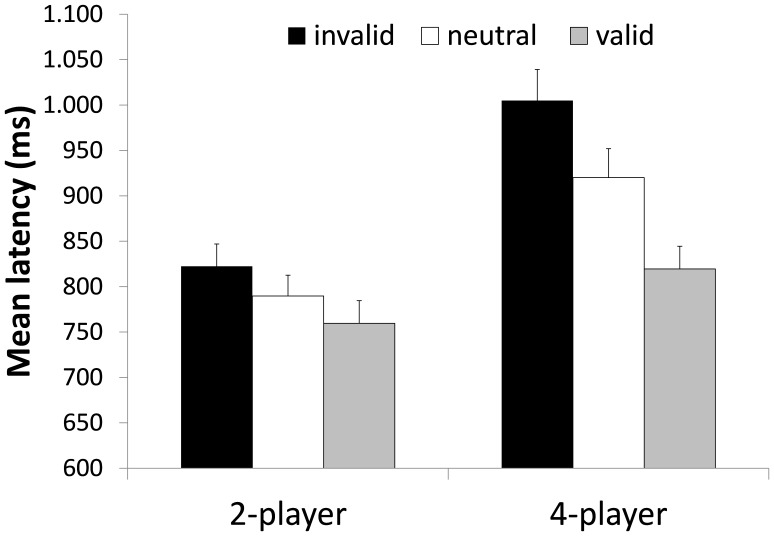
Mean response times of Experiment 1. RTs are depicted as a function of WM validity and set size of the task. Error bars indicate standard errors.

Trials were only included in the RT analysis if both the memory probe and the decision “who has the ball” were correct. The descriptive statistics of the RT over all participants of Experiment 1 are illustrated in Figure 2. The 2 (set size)×3 (validity) ANOVA revealed a main effect for set size *F*(1, 23) = 38.259, *p* = .000, *η^2^_p_* = .625 with faster RT in the 2 player condition. Most importantly and in support of the biased competition theory the 2×3 ANOVA revealed a main effect for validity *F*(1.493, 34.334) = 66.029, *p* = .000, *η^2^_p_* = .742.

Of particular interest, the set size×validity interaction *F*(2, 46) = 14.556, *p* = .000, *η^2^_p_* = .388 was significant. Planned contrasts revealed that the significant interaction was due to larger validity effects in the 4-player condition with all three contrasts–valid vs. invalid, neutral vs. invalid, and valid vs. neutral–showing larger effect sizes in the 4-player condition compared to the 2-player condition. For the 4-player condition the planned contrast showed significant differences between valid vs. invalid trials, *F*(1, 23) = 94.315, *p* = .000, *η^2^_p_* = .804; neutral vs. invalid trials *F*(1, 23) = 35.525, *p* = .000, *η^2^_p_* = .607; neutral vs. valid trials *F*(1, 23) = 33.349, *p* = .000, *η^2^_p_* = .592. Whereas, the effects were less pronounced in the 2-player condition: valid vs. invalid trials, *F*(1, 23) = 12.652, *p* = .002, *η^2^_p_* = .355, neutral vs. invalid trials *F*(1, 23) = 6.700, *p* = .016, *η^2^_p_* = .226, and neutral vs. valid trials *F*(1, 23) = 6.507, *p* = .018, *η^2^_p_* = .221.

#### Decision errors

**Figure 3:**
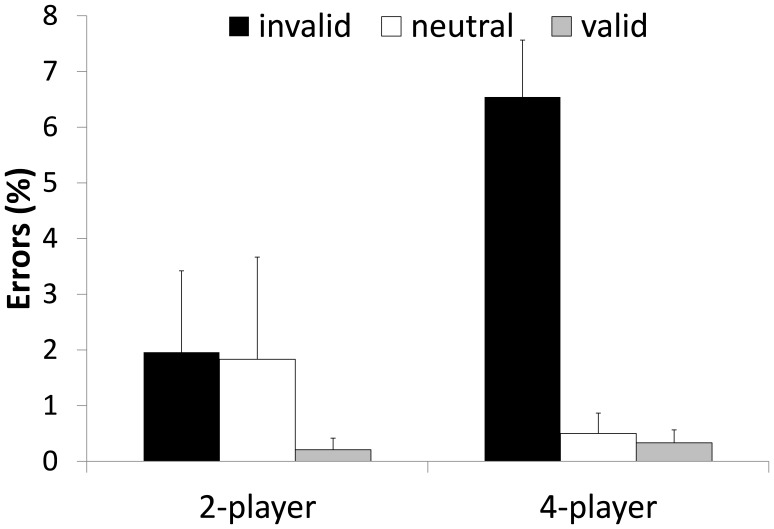
Mean errors in per cent of Experiment 1. Errors are depicted as a function of WM validity and set size of the task. Error bars indicate standard errors.

The descriptive statistics of the percentages of decision errors over all participants of Experiment 1 are illustrated in Figure 3. Although, we illustrate the direct proportion scores in Figure 3 we analyzed the log10 transformed error rates due to a skewed distribution and in order to avoid an overestimation of significance values. The 2 (set size)×3 (validity) ANOVA revealed a main effect for validity *F*(2, 46) = 26.747, *p* = .000, *η^2^_p_* = .538 with most errors in the invalid condition. The main effect of set size was also significant *F*(1, 23) = 18.121, *p* = .000, *η^2^_p_* = .441. As for the response time data the ANOVA showed a significant set size×validity interaction *F*(2, 46) = 21.752, *p* = .000, *η^2^_p_* = .486 as the contents of WM interfered more with response accuracy in the 4-player condition. While planned contrast on the log10 transformed error data in the 2-player condition did not reveal significant differences between the validity conditions (all *p*>.2), planned contrast in the 4-player conditions showed significant differences between valid and invalid trials, *F*(1, 23) = 60.096, *p* = .000, *η^2^_p_* = .723, between neutral and invalid trials *F*(1, 23) = 49.369, *p* = .000, *η^2^_p_* = .682, but not between neutral and valid trials *(p = *.71). Hence, the significant interaction was due to a substantial increase in error rates in the 4-player invalid condition. Figure 3 demonstrates this enhanced interfering effect from information in WM in the 4-player invalid condition.

### Discussion

The results of Experiment 1 supported hypothesis (a) by showing that attention was guided automatically by a template held in WM for both experienced athletes and novices. Of particular relevance the attentional guidance effect interacted significantly with set size and thereby supported hypothesis (b). By increasing the number of potential “ball-holders” in the experimental task participants were required to broaden their attentional window [30] in order to include all potential “ball holders” which in turn increased attentional guidance effects by WM as more objects were competing for attention.

Besides demonstrating attentional guidance from WM on the RT data the results from Experiment 1 further indicated that the contents of WM also influence the number of errors. This effect could not have necessarily been predicted from previous studies on attentional guidance from WM as these usually used simple discrimination tasks that did not result in sufficient error variance for analysis. By assigning different behavioral responses to the objects in the visual display the attentional guidance effect was also evident on error rates which indicates that once visual attention was drawn to an object participants were more likely to press the key assigned to that object even if it was not the target. In this respect the results of Experiment 1 go beyond studies reporting attentional guidance effects on RT data. Again, this effect was especially pronounced in the 4-player condition indicating that participants made more errors if more objects were competing for attention.

Based on the error data we return to the second question raised by Soto et al. [16] if the automatic capture of attention by WM can lead to some of the action errors that can occur in everyday situations. In this regard, we attempted to test if team sport athletes fail to pass to a unmarked player in a computer based sport task if they are holding a representation of a different player in WM.

## Experiment 2: “Who Should Get the Ball?”

In Experiment 2 we tested whether holding an image of a certain player in WM biases attention towards that player and thereby facilitates the decision to pass to this player. By experimentally manipulating whether this player is guarded or not guarded, we investigated attentional guidance from WM in a schematic computer based sport decision making situation.

Moreover, we attempted to corroborate the set size findings of Experiment 1. Specifically we hypothesized that (a) participants show the fastest RTs when they are holding an image of a certain cartoon player’s face in WM who is subsequently unmarked (valid trial) and therefore the best passing option. The slowest RTs should be evident when the player being held in WM is guarded (invalid trial) as attention will be automatically drawn towards him and subsequently attention has to be reallocated towards the open team-member. If the player being held in WM does not appear in the decision display (neutral trial) then RTs should fall in-between valid and invalid trials; (b) we hypothesized to find a similar pattern of decision errors as participants are time pressured when choosing a passing option in the computer based sports task and therefore should be prone to more impulsive errors in the invalid condition as compared to the neutral and valid condition; (c) in line with Experiment 1, we expected that players rely more on attentional guidance from WM when more passing opportunities compete for attention as compared to situations with fewer passing opportunities competing for attention; (d) we again explored differences between participants with no competitive team-sport experience and experienced team sport players.

### Method

#### Ethics statement

The study was approved by the Ethics board of the German Sport University Cologne. Informed consent was obtained from every participant before commencing the experiment. The study was carried out in accordance with the Helsinki Declaration of 1975. Before commencing the experiment participants filled out a questionnaire gathering biographic data.

#### Participants

Altogether 52 new participants (20 female; age *M* = 24.5) took part in the study. Neither age nor gender moderated the pattern of results in Experiment 2. Half of the participants (*n* = 26) were experienced Handball players (*M* = 14.2 years of competitive playing experience), whereas the other half had no competitive team-sports experience. The participants were unaware of the purpose of the study. All of the participants reported to have normal or corrected to normal vision. The participants volunteered to participate in the study and no kind of compensation was given for participation in the study.

#### Apparatus, task, and stimuli

**Figure 4:**
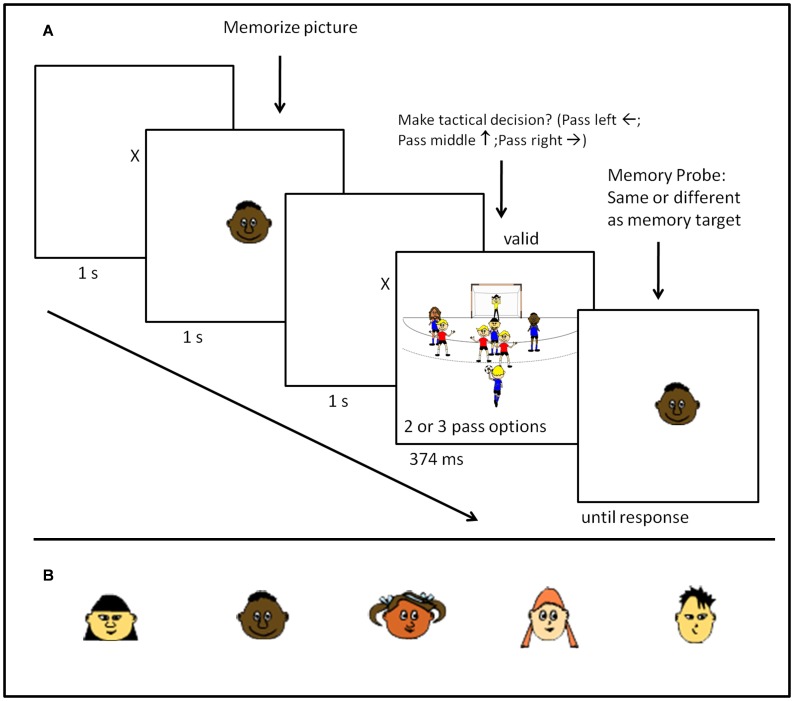
Task and stimuli utilized in Experiment 2. (A) Sequence of a sample trial in Experiment 2. (B) Heads of cartoon characters from the easy Sport-Graphic Software 2.0 Handball used as memory items in Experiment 2.

The apparatus was identical to Experiment 1. Figure 4A gives a schematic illustration of a sample trial of Experiment 2. Each experimental trial started with a fixation cross being presented for 1500 ms followed by the presentation of the visual memory item–a cartoon face of a player, as the target players did not differ in clothing as in Experiment 1–for 1000 ms. Participants were instructed to memorize the cartoon face as they had to identify it in a subsequent memory probe task. Responses and ratios of matching and mismatching faces in the memory probe were identical to Experiment 1. Another fixation cross appeared for 1500 ms after the memory item was presented, fixating the gaze of the participants exactly on the centre of the subsequent tactical decision making display. The decision display was the main difference to Experiment 1. This time participants did not have to identify which player had the ball in a structured visual display–all visual objects located on the same radius from fixation–but had to make a situation appropriate passing decision in a computer generated unstructured cartoon image that resembled a team handball situation. The stimulus array resembled the frontal perspective of a team handball court including the goal, the relevant court lines, the attacking players, the defending players, and the goal-keeper. In order to simulate the time pressure team sport players are confronted with when making tactical decisions, the decision array was only presented for 374 ms in Experiment 2.

Depending on the experimental condition there were either two (2-player condition) or three (3-player condition) identical looking defenders from the easy Sports-Graphics Software wearing red jerseys arranged around the goal area line. In the 2-player condition the two defenders could occupy three potential positions, whereas in the 3-player condition the three defenders could occupy 5 potential positions (cf. Figure 4A for a sample stimulus of the 3-player condition) always leaving one attacking player unguarded. The attacking players were drawn randomly from the pool of the 5 attacking players (all wearing identical blue jerseys with only their heads and skin color differing, cf. Figure 4B) assuring that every player occurred equally often in the respective experimental condition. Except for the attacking player holding the ball the offensive players faced the participant and their facial features were fully visible. In the 2-player condition the two potential passing options always occupied the same position at 16° radius to the left and right from fixation. In the 3-player condition the three players who could potentially receive the ball were either on fixation or 16° radius to the left or right from fixation. The participant’ s task was to identify as quickly and accurately as possible which player was unmarked and therefore would be the best passing option by pressing the corresponding arrow keys.

#### Procedure

In contrast to Experiment 1 we manipulated set size between subjects and only had one practice block consisting of 6 trials and one experimental block consisting of 72 trials. Fifteen experienced Handball players and 13 participants with no team sport experience participated in the 3-player version of the experiment, whereas 12 experienced Handball players and 12 participants with no team sport experience took the 2-player version. The practice block used exactly the same procedure as the following experimental block. Again both the memory object and the players in the decision array were selected randomly from the five cartoon figures displayed in Figure 4B. The ratio of valid, neutral, and invalid trials was again 33 per cent as in Experiment 1. Valid trials were characterized by the memory item being the same cartoon face as the face of the unguarded offensive player. On neutral trials the memory item was not present in the decision array and the two or three passing options had different identities compared to the memory item. On invalid trials the memory item was present in the decision array, but was guarded by a defensive player. Participants were instructed to respond as quickly and as accurately as possible on the decision task, whereas only accuracy was emphasized on the memory probe task.

#### Data analysis

Again there was neither a main effect nor any interactions for the factor handball experience on both RTs and errors (all *p*>.24) which were almost identical. Thus, we did not further differentiate between handball experiences as in Experiment 1. We ran two mixed design ANOVAs with the between subject independent variable set size and the within subject independent variable validity on both response times and the log10 transformed decision errors. Where, the assumption of sphericity was violated, the p-values for main effects were computed using the conservative Greenhouse-Geisser method with corrected degrees of freedom. We followed up significant main effects and interactions with planned contrasts.

### Results

Performance on the memory task was good (*M* = 93.3% correct, *SD* = 7.4) and demonstrated that participants were holding the object in WM which is essential for testing the predictions of BCT.

#### Response times (RT)

**Figure 5:**
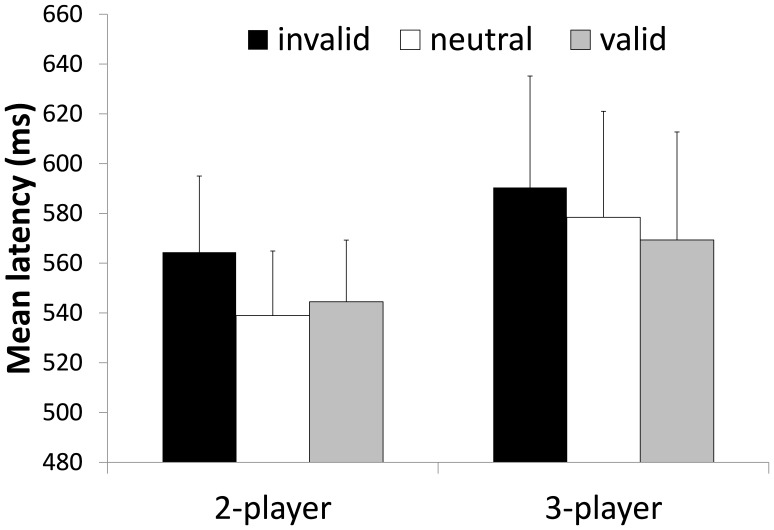
Mean response times of Experiment 2. RTs are depicted as a function of WM validity and set size of the task. Error bars indicate standard errors.

Trials were only included in the RT analysis if both the memory probe and the decision “who to pass the ball to” were correct. The descriptive statistics over all participants of Experiment 2 are illustrated in Figure 5. The 2 (2-player/3-player)×3 (invalid/neutral/valid) mixed design ANOVA only revealed a main effect for validity *F*(2, 100) = 8.195, *p* = .001, *η^2^_p_* = .141. No other main effects (set size: *p* = .57) nor interactions (set size×validity: *p* = .35) were evident. Planned contrasts were run to explain the main effect of validity which revealed significant differences between valid and invalid trials, *F*(1, 51) = 12.652, *p* = .001, *η^2^_p_* = .199, between neutral and invalid trials *F*(1, 51) = 11.936, *p* = .001, *η^2^_p_* = .190, but not between neutral and valid trials *(p = *.69).

#### Decision errors

**Figure 6:**
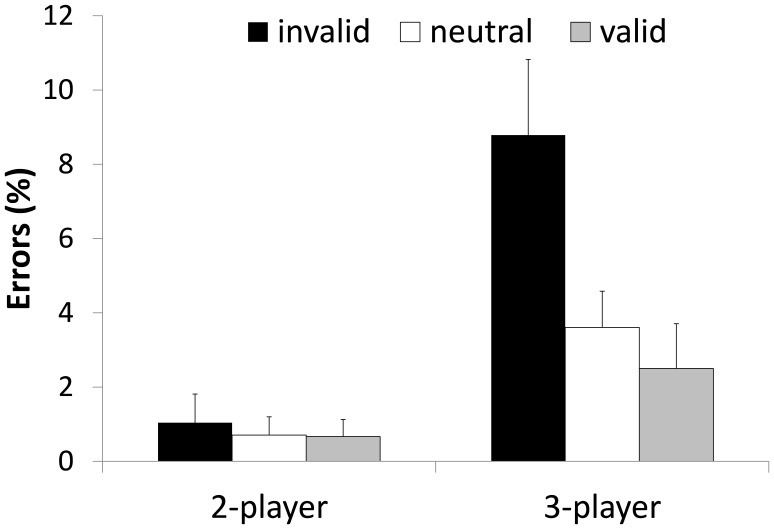
Mean errors in per cent of Experiment 2. Errors are depicted as a function of WM validity and set size of the task. Error bars indicate standard errors.

The descriptive statistics of the percentages of decision errors over all participants of Experiment 1 are illustrated in Figure 6. The 2 (2-player/3-player)×(invalid/neutral/valid) mixed design ANOVA revealed a main effect for set size, *F*(1, 50) = 14.938, *p* = .000, *η^2^_p_* = .230), and validity, *F*(1.757, 87.833) = 8.593, *p* = .001, *η^2^_p_* = .147 with more errors in the 3-player and the invalid conditions. Further, the ANOVA showed a significant set size×validity interaction, *F*(1.757, 87.833) = 7.232, *p* = .002, *η^2^_p_* = .126 as the contents of WM interfered more with response accuracy in the 3-player condition. Similar to Experiment 1 planned contrast on the log10 transformed error data in the 2-player condition did not reveal significant differences between the validity conditions (all *p*>.41), whereas planned contrast in the 3-player conditions showed significant differences between valid and invalid trials, *F*(1, 27) = 16.742, *p* = .000, *η^2^_p_* = .383, between neutral and invalid trials *F*(1, 27) = 10.424, *p* = .003, *η^2^_p_* = .279, but not between neutral and valid trials *(p = *.09). Hence, the significant interaction was due to a substantial increase in error rates in the 3-player invalid condition (cf. Figure 6).

### Discussion

In Experiment 2 we were able to provide first support for hypothesis (a) that attentional guidance from WM also occurs in a schematic sports situation. Hence, the pattern of results from Experiment 2 suggests that if a team-sport athlete holds a representation of a certain player in WM then his or her attention will be automatically drawn towards this player and thereby facilitates the decision to pass to this player. In situations in which this player is unmarked the attentional guidance effect from WM is beneficial (valid trials), whereas it is detrimental in situations in which the player is guarded as attention is drawn to this player automatically and subsequently has to be reoriented towards a more suitable passing opportunity. This pattern of results is evident both in the RT data and in the error rate data. Hence, we also confirm hypothesis (b) that holding a representation of a certain player in WM does not only influence attentional orienting but also the actual decision of whom to pass to. Experiment 2 only partially confirmed hypothesis (c). This time data only demonstrated a set size×validity interaction on the error data and not on the RT data.

The general pattern of results supports our assumption that a template being held in WM can result in a pass to a guarded team-member in a schematic computer based sport task which in turn might lead to a turnover. Understanding more about suboptimal passing decisions has important applied implications as these often result in turnovers in team sports such as basketball or interceptions in American football that have empirically been linked to the success of teams, especially in close games [37]. For example in basketball successful teams have greater passing skills (more successfully completed passes) and less turnovers [38], [39]. Therefore, we attempted to move our experimental paradigm further along the “artificial/naturalistic” continuum [26] by creating a new task consisting of photo images of 2 vs. 1 and 3 vs. 2 basketball training situations in which participants had to decide which player to pass to.

## Experiment 3: “Whom Should I Pass To?”

**Figure 7:**
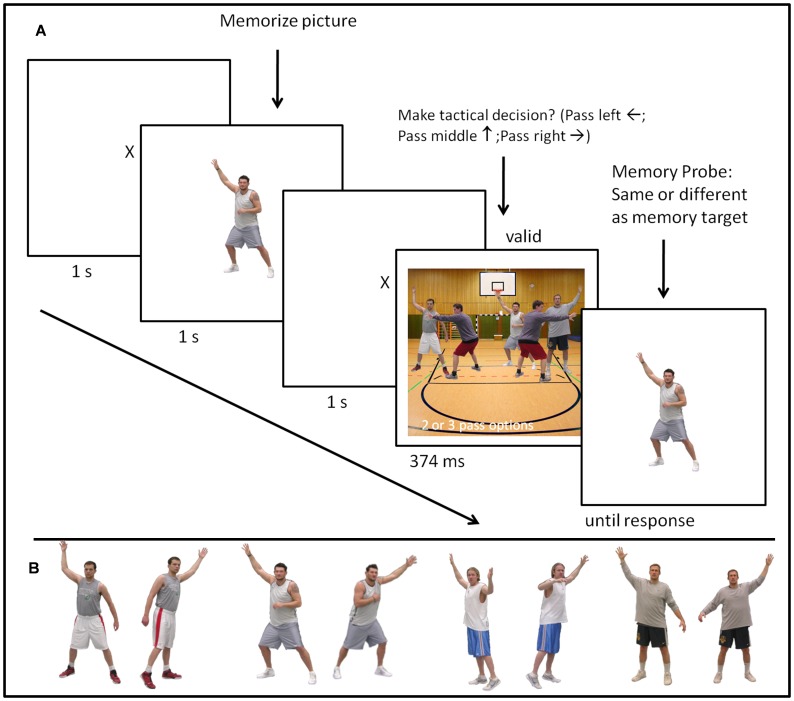
Task and stimuli utilized in Experiment 3. (A) Sequence of a sample trial in Experiment 3. (B) Photographs of the basketball players used as memory items in Experiment 3. All actors provided written informed consent for publication of the images.

The purpose of Experiment 3 was to test the assumptions of BCT using naturalistic photo stimuli of a sport context (cf. Figure 7). We tested the same predictions derived from BCT as in Experiment 2. In our endeavour of ”grounding our task in the real-life experience” of basketball we further implemented two different viewing distances as a decision maker in basketball will not always be viewing the exact same scenario. An additional rationale for implementing these two different viewing condition was investigating if the attentional guidance effect only occurs if the memory item perfectly matches the visual object in the stimulus array in size or also occurs to a similar degree if the size of the memory item is different as the size of the visual object in the decision array. In addition, we attempted to scrutinize the null findings from Experiment 1 and 2 concerning team-sport experience in a task using photo snap shots of a basketball situation.

### Method

#### Ethics statement

The study was approved by the Ethics board of the German Sport University Cologne. Informed consent was obtained from every participant before commencing the experiment. The study was carried out in accordance with the Helsinki Declaration of 1975. Before commencing the experiment participants filled out a questionnaire gathering biographic data.

#### Participants

Altogether 40 new participants (20 female; age *M* = 23.55) took part in the study. Neither age nor gender moderated the pattern of results in Experiment 3. Half of the participants (*n* = 20) were experienced basketball players (*M* = 8.43 years of competitive basketball experience), whereas the other half had no competitive team sport experience. The participants were unaware of the purpose of the study. All of the participants reported to have normal or corrected to normal vision. The participants volunteered to participate in the study and no kind of compensation was given for participation in the study. Informed consent was obtained from every participant before commencing the experiment. The study was carried out in accordance with the Helsinki Declaration of 1975.

#### Apparatus, task, and stimuli

The apparatus was identical to Experiment 1 and 2. Figure 7 gives a schematic illustration of a sample trial of Experiment 3. The task was almost identical to Experiment 2 and therefore only the changes are described. In Experiment 3 the visual memory item was a photograph of an attacking basketball player awaiting to receive a pass (cf. Figure 7B). Otherwise, the decision display was the only further difference compared to Experiment 2. This time participants did not have to decide to which cartoon figure a certain players should pass to in a schematic handball scenario but had to decide to which player they would pass to in a basketball situation photographed from the first person perspective. Again, depending on the experimental condition there was either one (2-player condition) or two (3-player condition) defenders present in the stimulus display. The defender was always the same person wearing a green basketball training jersey photographed in different representative basketball defensive positions. In the 2-player condition the defender could occupy two potential positions (guarding either the left attacking player or the right attacking player), whereas in the 3-player condition the two defenders could occupy 3 potential positions (guarding either the left and the right attacking player, or the middle player with either the left or right player) always leaving one attacking player unguarded. The attacking players were drawn randomly from the pool of the 4 attacking players (once photographed in a simulated basketball posture when awaiting a pass on the right side and once from the left side) assuring that every player occurred equally often in the respective experimental condition. The attacking players all wore light colored basketball training jerseys that were not exactly identical to each other (cf. Figure 7B) which is common procedure in basketball training situations.

We did not include a neutral trial in Experiment 3, as it is fairly unlikely that a point-guard in basketball would form the intention to pass to a certain player that is not present on the court. Moreover as stated above, we implemented two different viewing distances in order to assess whether the attentional guidance effect only emerged when the memory item perfectly matched the attacking player in the subsequent decision display. Thus, the decision array was build up from two different basketball court templates with half the trials simulating the decision maker being further away from the basket (far) and therefore the players appearing further away and the other half simulating the decision maker being closer (close) and therefore the players appearing closer. In the close viewing condition the size of the offensive players was approximately (not absolutely identical as the individual players differed in size) 14.3°×5.82° radius in the decision display and was identical to the memory display. In the close condition both the left and the right passing options were 16° radius from fixation in the 2-player condition, whereas in the 3-player condition the third potential passing option was at fixation. In the far condition the size of the offensive players was 10.5°×4.77° radius and was therefore reduced in size in the decision array compared to the memory array. In the far condition both the left and the right passing options were 19° radius from fixation in the 2-player condition, whereas in the 3-player condition the third potential passing option was at fixation.

#### Procedure

The procedure of Experiment 3 was similar to Experiment 2. This time we chose to manipulate set size within participants and had two experimental blocks that were administered in random order. The two experimental blocks consisted of 48 trials. The 3-player block involved three passing options–left, middle, or right–whereas the 2-player block only involved two passing options–left or right. The ratio of valid/invalid trials was 50 per cent. Each experimental block was preceded by a practice block consisting of 8 practice trials–2 close/valid, 2 far/valid, 2 close/invalid, 2 far/invalid. Again both the memory object and the players in the decision array were selected randomly from the pool of characters displayed in Figure 7B assuring that each character was selected equally often. Participants were instructed to respond as quickly and as accurately as possible on the decision task, whereas only accuracy was emphasized on the memory probe task.

#### Data analysis

Again the data for both participant groups was almost identical and there was neither a main effect nor any interactions (all p>.3) for the factor basketball experience on both RTs and errors. Thus, we did not further differentiate between experienced basketball players and novices in Experiment 3. We ran two-factor ANOVAs with repeated measures on both within subject independent variables (validity and set size) on both response times and log10 transformed decision errors.

### Results

Performance on the memory task was good (*M* = 94.3% correct, *SD* = 3.7) and demonstrated that participants were holding the object in WM which is essential for testing the predictions of BCT. No differences were evident between the close and the far stimuli for both RTs (*p* = .37) and errors (*p* = .20) and we therefore did not treat the viewing conditions as an additional factor in the data analysis.

#### Response times (RT)

**Figure 8:**
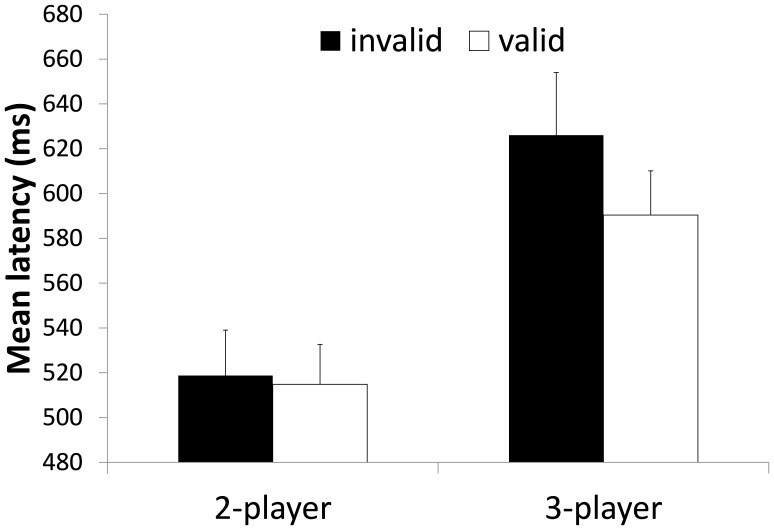
Mean response times of Experiment 3. RTs are depicted as a function of WM validity and set size of the task. Error bars indicate standard errors.

Trials were only included in the RT analysis if both the memory probe and the decision “whom to pass the ball to” were correct. The descriptive statistics over all participants of Experiment 3 are illustrated in Figure 8. The 2 (2-player/3-player)×2 (invalid/valid) within subject ANOVA revealed a main effect for validity (*F*(1, 39) = 4.660, *p* = .037, *η^2^_p_* = .109) with faster RTs in valid trials compared to the invalid condition. The ANOVA further revealed a main effect for set size *F*(1, 39) = 34.088, *p* = .000, *η^2^_p_* = .473 with faster response times in the 2-player condition. The set size×validity interaction did not reach significance *F*(1, 39) = 1.878, *p* = .179, *η^2^_p_* = .047 but showed a tendency towards greater attentional guidance findings in the 3-player condition. Although, the interaction was not significant we followed up the ANOVA with two separate pairwise comparisons that only revealed a significant differences between valid and invalid trials in the 3-player condition (*p* = .038, one-tailed) but not in the 2-player condition (*p* = .18, one-tailed).

#### Decision errors

**Figure 9:**
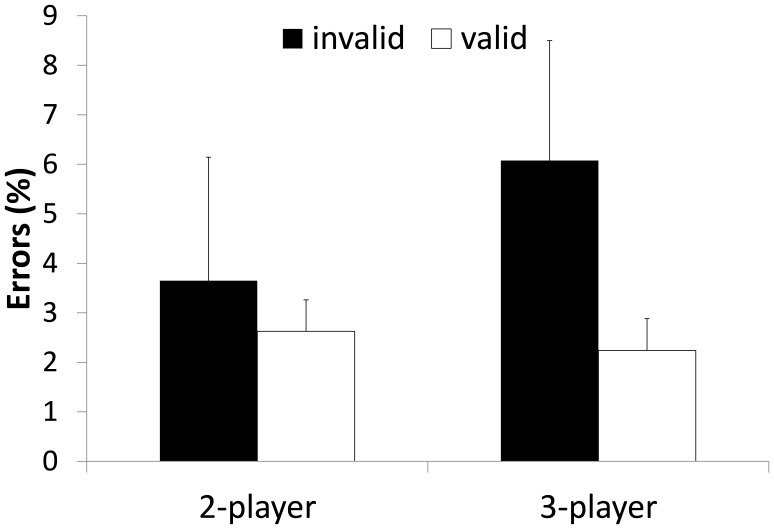
Mean errors in per cent of Experiment 3. Errors are depicted as a function of WM validity and set size of the task. Error bars indicate standard errors.

The descriptive statistics of the percentages of decision errors over all participants of Experiment 3 are illustrated in Figure 9. The 2 (2-player/3-player)×2 (invalid/valid) within subject ANOVA on decision errors only revealed a main effect for validity, *F*(1, 39) = 4.946, *p* = .032, *η^2^_p_* = .113, showing fewer errors in the valid condition compared to the invalid condition. No main effect for set size, *F*(1, 39) = .894, *p* = .350, *η^2^_p_* = .022, was evident. However, and in line with Experiments 1 and 2 the interaction between set size and validity was significant, *F*(1, 39) = 11.241, *p* = .002, *η^2^_p_* = .224, demonstrating that attentional guidance had a greater effect in the 3-player condition (cf. Figure 9). Follow up pairwise comparisons only revealed a significant differences between valid and invalid trials in the 3-player condition (*p* = .000, one-tailed) but not in the 2-player condition (*p* = .25, one-tailed).

### Discussion

The results from Experiment 3 confirm the findings from Experiment 1 and 2 by showing the attentional guidance effect from WM with naturalistic team-sport photos. Hence, an athlete’s attention was biased in a computer based basketball task towards certain team-members that resemble internal templates that are currently being held in WM. In this respect, we argue that this might be similar to the common situation in team-sports in which players form an intention to pass to a certain player and this player therefore receives a competitive advantage over other players in the visual field due to biased attention which does not only influence response times but also the actual decision with more passing errors in the invalid condition. This effect increases with more visual objects in a certain situation as there is more competition to be resolved between visual objects competing for limited attention resources [10].

Moreover, the results again did not differ between experienced basketball players and participants with no competitive basketball experience. Even when artificially combining the data from Experiment 2 and 3–as far as possible due to important differences in the design (within vs. between manipulation of set size, 2 vs. 3 levels of the independent variable validity): There was no main effect of team-sport experience when combining Experiment 1 and 2 in the large set size condition F(1, 66) = .359, p = .551, η^2^p = .005, nor an 2 (invalid vs. valid)×2 (team-sport experience vs. no team sport experience) interaction on the reaction time data F(1, 66) = 1.173, p = .283, η^2^p = .017. The ANOVA only showed a main effect for validity F(1, 66) = 6.110, p = .016, η^2^p = .085. The same pattern emerged on the error data: No main effect of team sport experience (F(1, 66) = .054, p = .817, η^2^p = .001), main effect of validity (F(1, 66) = 11.931, p = .001, η^2^p = .153), and no 2 (invalid vs. valid)×2 (team-sport experience vs. no team sport experience) interaction (F(1, 66) = 2.409, p = .125, η^2^p = .035). Team sport experience also did not have any effect in the small set size condition.

## General Discussion

The main aim of the studies was to transfer BCT to the field of sports and follow the call of Kingstone et al. [22] to replicate previous findings from basic attentional laboratory paradigms in tasks grounded in real-life experience. In this endeavor we gradually modified an existing experimental paradigm to resemble the sport situation of a player making a passing decision.

Besides replicating previous research in a task grounded in sports–i.e. making a passing decision while previously having formed the intention to pass to a certain player–which according to Kingstone et al. [22] is not a trivial research step but a research necessity [34], [35] especially in attentional research [40] the present research extends previous findings on BCT. First, the attentional guidance effect was not only evident in the RT analysis but also on errors in the decision task which suggests that once visual attention has been drawn towards a memory matching object this object also receives a competitive advantage in influencing behavior. A further important extension to previous research was that the attentional guidance effect from WM increased if more objects in the visual scene competed for limited attentional resources. This result might be explained by a similar line of research that reported greater attentional guidance effects from WM when participants increased their attentional window in a visual search task [30]. Hence, we argue that including more potential passing opportunities requires participants to adopt a broader attentional window which results in greater attentional guidance effects as there is more competition to be resolved between decision options competing for limited attentional capacity.

Despite these novel contributions to the cognitive attention literature the conducted studies have their limitations in explaining sports performance. Computer-based research paradigms have been criticized as providing limited insight in understanding sport performance, especially due to poor stimulus response compatibility [41], [42] and therefore the practical implications of the present findings are not clear. Although, the present research paradigm was derived from the everyday sport experience of a decision maker forming the intention of passing to a certain team-member–which we argue resembles the general cognitive mechanism of how the activated contents in WM control the visual focus of attention–future research needs to extend our first findings on the BCT in dynamic representative sport contexts. A potential approach in this endeavor might be derived from [43]. This ecological approach to cognition in sport criticizes an overemphasis on inner processes in sport psychology while arguing that human behavior cannot be understood in isolation of the environment in which it occurs.

Within cognitive psychology a further promising approach–cognitive ethology [44]–has recently been put forth due to converging evidence that cognitive processes substantially depend on the situational context in which a person is embedded. In a nutshell the cognitive ethology approach [44] sees real world and lab-based investigations as complementary in that the researcher should first systematically observe what is naturally occurring and then apply the rigorously controlled lab-based approach to evaluate and experimentally test the real-world observation. A recent review [26] suggests that research programs applying this approach have revealed central cognitive mechanisms in tasks varying in their approximation to real life but also important differences. Therefore, future research on BCT has to move further along the continuum towards more naturalistic sport performance contexts and test our first findings on biased competition from WM in a representative sport context. This approach will reveal whether attentional guidance from WM can be considered a central cognitive mechanism involved in the allocation of attention in sport as suggested by the present laboratory experiments.

Despite the need for more representative research designs in examining attentional processes in sports, we consider the concept of WM as a promising guiding framework for deriving testable hypothesis, as sport attention research remains underdeveloped and has been criticized [45] of lacking a theoretical framework: “a suitable framework to study the influence of attention on sport skills has not been established” (p. 326). This argument is based on the frequent statement in the cognitive literature that the WM framework is useful for studying attention in complex everyday behavior [4], [10], [46], [47].

Finally, the null-finding concerning specific sport practice requires discussion. As sport specific experience did not influence the pattern of results it seems as if the attentional guidance effect from WM is not influenced by sport-specific experience. However, this conclusion is not warranted at present and several alternative interpretations are feasible. First, following the above argumentation the experimental task might not have tapped the domain of expertise of the athletes and therefore no differences emerged on the computer based sport task as a large body of evidence [32] suggests that expert-novice differences only emerge on tasks directly related to the respective fields of expertise [48], [49]. A further confounding variable that was not controlled for in the present study is physical fitness. Recent research [50], [51] has demonstrated enhanced cognitive functioning as a consequence of increased physical fitness. Although, it is likely that our athlete group possessed superior aerobic fitness than the non-athlete group it is possible that both groups had equal levels of aerobic fitness which might have accounted for the null effect on the cognitive task. Hence, future research should address these shortcomings in order to provide a stronger case on the moderating effect of sport specific experience on attentional guidance from WM.

In conclusion, BCT theory has the potential to be a valuable framework for guiding research and deriving testable hypothesis, also in applied settings such as sports. The results suggest that certain decision options receive a competitive advantage if they are associated with the activated contents in the circuitry of WM and that this effect is especially pronounced in situation with several decision options competing for attention.
